# Correction: Assessment of Competition between Fisheries and Steller Sea Lions in Alaska Based on Estimated Prey Biomass, Fisheries Removals and Predator Foraging Behaviour

**DOI:** 10.1371/journal.pone.0135431

**Published:** 2015-08-05

**Authors:** The PLOS ONE Staff

## Notice of Republication

This article was republished on July 23, 2015, to correct errors in Fig 5–10 that were introduced during the production process. The publisher apologizes for the errors. Please download this article again to view the correct version. The originally published, uncorrected article and the republished, corrected article are provided here for reference.

## Supporting Information

S1 File Originally published, uncorrected article. (PDF)

S2 File Republished, corrected article. (PDF)
