# Supplementary material for: Correction: Assessment of Competition between Fisheries and Steller Sea Lions in Alaska Based on Estimated Prey Biomass, Fisheries Removals and Predator Foraging Behaviour
Source: PLoS One. 2015 Aug 5;10(8):e0135431. doi: 10.1371/journal.pone.0135431 (PMC4526339; doi:10.1371/journal.pone.0135431)
Supplement: S2 File — (PDF) [file pone.0135431.s002.pdf]

PLOS ONE

RESEARCH ARTICLE

Assessment of Competition between Fisheries and Steller Sea Lions in Alaska Based on Estimated Prey Biomass, Fisheries Removals and Predator Foraging Behaviour

Tabitha C. Y. Hui[¤a]*, **Rowenna Gryba**[¤b], **Edward J. Gregr**[¤c], **Andrew W. Trites**

Marine Mammal Research Unit, Fisheries Centre, University of British Columbia, Vancouver, B.C., Canada

¤a Current address: National Parks Board, National Biodiversity Centre, Singapore, Singapore,
¤b Current address: Stantec, Burnaby, B.C., Canada,
¤c Current address: Institute for Resources, Environment, and Sustainability, University of British Columbia, Vancouver, B.C., Canada
* tabitha.hui@gmail.com

OPEN ACCESS

Citation: Hui TCY, Gryba R, Gregr EJ, Trites AW (2015) Assessment of Competition between Fisheries and Steller Sea Lions in Alaska Based on Estimated Prey Biomass, Fisheries Removals and Predator Foraging Behaviour. PLoS ONE 10(5): e0123786. doi:10.1371/journal.pone.0123786

Academic Editor: Antoni Margalida, University of Lleida, SPAIN

Received: July 3, 2014

Accepted: March 8, 2015

Published: May 7, 2015

Copyright: © 2015 Hui et al. This is an open access article distributed under the terms of the Creative Commons Attribution License, which permits unrestricted use, distribution, and reproduction in any medium, provided the original author and source are credited.

Data Availability Statement: All relevant data are within the paper.

Funding: Financial support was provided by National Oceanic and Atmospheric Administration (NOAA) to the North Pacific Universities Marine Mammal Research Consortium through the North Pacific Marine Science Foundation. The funders had no role in study design, data collection and analysis, decision to publish, or preparation of the manuscript.

Competing Interests: The authors have declared that no competing interests exist.

Abstract

A leading hypothesis to explain the dramatic decline of Steller sea lions (*Eumetopias jubatus*) in western Alaska during the latter part of the 20th century is a change in prey availability due to commercial fisheries. We tested this hypothesis by exploring the relationships between sea lion population trends, fishery catches, and the prey biomass accessible to sea lions around 33 rookeries between 2000 and 2008. We focused on three commercially important species that have dominated the sea lion diet during the population decline: walleye pollock, Pacific cod and Atka mackerel. We estimated available prey biomass by removing fishery catches from predicted prey biomass distributions in the Aleutian Islands, Bering Sea and Gulf of Alaska; and modelled the likelihood of sea lions foraging at different distances from rookeries (accessibility) using satellite telemetry locations of tracked animals. We combined this accessibility model with the prey distributions to estimate the prey biomass accessible to sea lions by rookery. For each rookery, we compared sea lion population change to accessible prey biomass. Of 304 comparisons, we found 3 statistically significant relationships, all suggesting that sea lion populations increased with increasing prey accessibility. Given that the majority of comparisons showed no significant effect, it seems unlikely that the availability of pollock, cod or Atka mackerel was limiting sea lion populations in the 2000s.

Introduction

Commercial fishing is assumed to be a contributing factor in the decline of Steller sea lions (*Eumetopias jubatus*) in Alaska because of the concurrent expansion of groundfish fisheries for walleye pollock (*Theragra chalcogramma*), Pacific cod (*Gadus macrocephalus*) and Atka

mackerel (*Pleurogrammus monopterygius*) [1–8]. These fisheries targeted fish of similar size and age as those eaten by sea lions [9–12], and had the potential to decrease sea lion foraging efficiency by altering the abundance, composition and distribution of the available prey field. Reduced prey availability could have resulted in a diet that was insufficient to meet the energy requirements of sea lions [8,13].

Loughlin and Merrick [4], Trites and Larkin [14], Ferrero and Fritz [15], Sampson [16], Trites et al. [17], Dillingham et al. [18], Hennen [19] and Calkins [20] have all tried to detect an effect of commercial fisheries on Steller sea lion populations. All have identified some correlations between catches and sea lion population declines, but the correlations have tended to be inconsistent with the overall patterns of sea lion declines, and have been specific to particular periods, geographic regions, prey species and gear types.

In addition to reducing the overall levels of prey biomass, fisheries can also remove or disperse large aggregations of fish from an area [21–23]. Such localised depletion (intense fishing pressure leading to disproportionately large reductions in local densities of the target fish relative to the overall harvest rate) could have negatively impacted sea lions by reducing foraging efficiency [24].

Assessing fisheries impacts on sea lions requires an estimate of localised prey abundance [25], particularly around rookeries and haulouts where sea lions rest and breed [14]. The broad-scale estimates of total prey abundance typically used in studies of potential competition between sea lions and fisheries [e.g., 26,27] may have little relevance to the prey available to foraging sea lions since the entire prey population is unlikely to be available to sea lions given its spatial and temporal distribution. Any competition between sea lions and fisheries is therefore more likely to occur and be detected on a local level than at a broad scale.

Accessibility (the likelihood of a sea lion foraging in a particular location near its terrestrial resting or breeding sites) is inversely correlated with the distance from shore. Satellite telemetry suggests that prey located closer to shore are likely more critical to the survival of sea lions than prey located further away [28–34]. Thus, it is important to determine the local accessibility and abundance of prey at varying distances from rookeries and haulouts to accurately assess correlations between fisheries and sea lions.

We sought to determine whether there was a relationship between available prey, commercial catch, and the rates of sea lion population change at 33 major rookeries (breeding sites) in western Alaska. We assumed that any effects of fishing that existed ought to be detectable as an effect of prey availability on sea lion population growth rate. Following the recommendations of Conn et al. [25], we sought to improve on previous studies of competition between sea lions and commercial fisheries by estimating the local (i.e., rookery scale) distribution and abundance of sea lion prey using continuous predictions of prey biomass for three commercially important fish species that have dominated the sea lion diet: walleye pollock (*Theragra chalcogramma*), Pacific cod (*Gadus macrocephalus*) and Atka mackerel (*Pleurogrammus monopterygius*) [35]. We tested for relationships between regional rates of sea lion population change, considering both pups and adults as suggested by Conn et al. [25], and the biomass of prey (pollock, cod or mackerel) accessible to sea lions during summer (2000–2008) with and without accounting for fishery removals. We also compared total biomass of prey removed annually by fisheries within a particular distance of each rookery and the annual rates of sea lion population change.

Methods

We calculated the annual change in Steller sea lion pups (<1 year old) and non-pups (>1 year old) from 2000 to 2008 at the 33 selected rookeries from population models developed by

Winship and Trites [36] and Battaile and Trites (supplementary data will be provided if manuscript is accepted) based on aerial and ground census counts made at rookeries by the U.S. National Marine Fisheries Service (NMFS). These models controlled for surveys conducted at different times of the day (i.e., whether most adult sea lions were at sea or on land, and when all juveniles were at sea or on land). We used the results from these models rather than the census counts themselves to account for missing survey years (i.e., pups: 2000, 2006–2008; non-pups: 2001, 2003, 2005) and to smooth out noise in the count data attributable to observation error.

We obtained catches of pollock, cod and mackerel from the North Pacific Groundfish Observer Program (NPGOP-NMFS) and models of habitat suitability and NMFS bottom trawl survey data [37] which predicted the spatial distribution of these species. Our study area included the Aleutian Islands, Bering Sea and Gulf of Alaska. We organized all data on a 9 x 9 km^2 grid in the Alaska Albers projection (NAD27) using ArcGIS 9.2 (ESRI, Redlands, CA) and IDRISI Kilimanjaro. We used R 2.8.1 [38] and the nlme library from Pinheiro and Bates [39] for all statistical analyses.

Sea lion population sizes and trends

We estimated population trends at each of the 33 major sea lion rookeries from the declining western stock of Steller sea lions (Fig 1)- 15 west and 18 east of Samalga Pass (hereafter referred to as Aleutian Island and Gulf of Alaska rookeries, respectively). We further partitioned the Aleutian Island rookeries into those east and west of Amchitka Pass, and the Gulf of Alaska rookeries into those east and west of Unimak Pass (Fig 1). The Sea Lion Rock (Amak) rookery was grouped with the western Gulf of Alaska rookeries because sea lions from this rookery likely forage in habitat similar to the other western Gulf of Alaska rookeries. We chose Amchitka, Samalga and Unimak Passes as regional breaks between the rookeries because of the known biological and oceanographic differences between these regions [40–45].

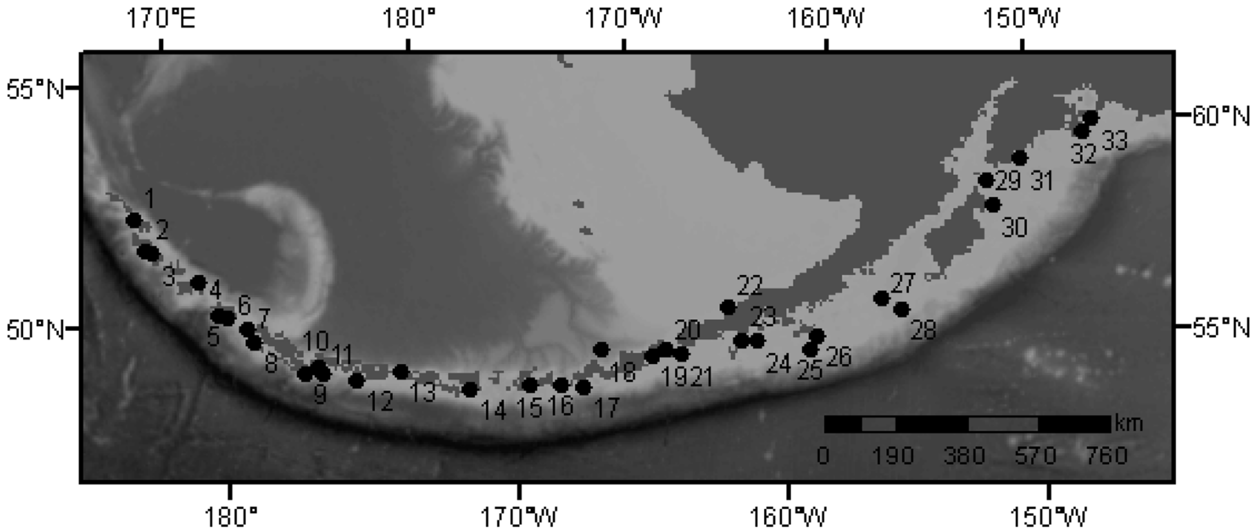

Fig 1. The locations of the 33 Steller sea lion rookeries studied. (1) Attu Cape Wrangell (2) Agattu Gillon Point (3) Agattu Cape Sabak (4) Buldir (5) Kiska Cape St Stephen (6) Kiska Lief Cove (7) Ayugadak (8) Amchitka Column Rock (9) Ulak Hasgox Point (10) Tag (11) Gramp Rock (12) Adak Lake Point (13) Kasatochi North Point (14) Seguam Saddle Ridge (15) Yunaska (16) Adugak (17) Ogchul (18) Bogoslof Fire Island (19) Akutan Cape Morgan (20) Akun Billings Head (21) Ugamak Round (22) Sea Lion Rock Amak (23) Clubbing Rocks North (24) Pinnacle Rock (25) Chernabura (26) Atkins (27) Chowiet (28) Chirikof (29) Sugarloaf (30) Marmot (31) Outer Pye (32) Wooded Fish (33) Seal Rocks. Rookeries were grouped into 4 regions (western Aleutian Islands—(1)-(8), eastern Aleutian Islands—(9)-(15), western Gulf of Alaska—(16)-(22), eastern Gulf of Alaska—(23)-(33)).

doi:10.1371/journal.pone.0123786.g001

Prey biomass distributions

We used predictions of catch per unit effort (CPUE) [37] (e.g., Fig 2) to calculate sea lion prey fields. Derived from NMFS bottom trawl survey data, these CPUE predictions were based on the relationship between surveyed CPUE and environmental data at a 9 x 9 km^2 resolution. Predictions were generated for years when surveys were conducted. This included 2000, 2002 and 2004 for pollock, cod and mackerel in the Aleutian Islands; and 2001 and 2003 for pollock and cod in the Gulf of Alaska and Bering Sea.

We averaged the June and July CPUE distributions for each year to form a summer CPUE distribution. We then distributed the total NMFS biomass estimates of pollock, cod and mackerel for the summer of each year throughout the respective fisheries survey areas in proportion to the CPUE within each grid cell. This formed a summer prey biomass distribution for each year. The biomass predictions were limited to depths less than 600 m—the known limits in the distribution of the fish species.

Fisheries catch

NPGOP-NMFS places trained observers on commercial fishing boats to monitor the quantity and composition of commercial catches. Although observer coverage on vessels $<$ 38.1 m is intermittent, vessels $>$ 38.1 m are required to have observers on board 100% of the time. The majority of pollock, cod and mackerel were caught by vessels $>$ 38.1 m [46]. Data recorded for pollock, cod and mackerel caught from 2000–2004 included the location and estimated biomass in each haul and CPUE.

Fisheries reduced prey biomass distributions

We created three different fishery-reduced prey distribution scenarios representing different hypotheses about how fisheries removals may cause localised depletions. First, we deducted the monthly (June and July) catches of pollock, cod and mackerel from the predicted summer prey biomass (average of June and July distributions) for each year, assuming the prey distribution did not change significantly between June and July (Scenario 1, e.g., Fig 2). For Scenario 2, we assumed that the distribution of prey biomass in July was the same as in June, and removed the cumulative biomass of pollock, cod and mackerel caught in June and July from the June prey biomass distributions of each year. Scenarios 1 and 2 assumed fishing removals caused a local reduction in fish abundance in the immediate vicinity of fishing and that this reduction remained geographically stable over June and July. The scenarios are distinct because the first is an average of the June and July distributions, while the second assumes that the distribution of fish in July was what remained following fishing in June.

For Scenario 3, we assumed that the total biomass of groundfish redistributed itself after each fisheries removal. We therefore deducted the biomasses of pollock, cod and mackerel caught in June and July from the respective trawl survey biomass estimates of each year, and distributed the resulting fisheries-reduced prey biomasses throughout the corresponding fisheries survey areas (Aleutian Islands, Eastern Bering Sea or Gulf of Alaska) in proportion to the predicted July CPUE distributions. Scenario 3 assumed that fishing reduced the overall biomass of prey available, but had little effect on the local availability of prey. All three methods of removing catch were intended to predict the biomass distributions of pollock, cod and mackerel at the end of July, after accounting for the total summer (June and July) catch.

Accessibility model

The accessibility of prey to Steller sea lions decreases with distance from a sea lion's terrestrial resting place and is critical for determining the impact of fisheries removal. We used at-sea

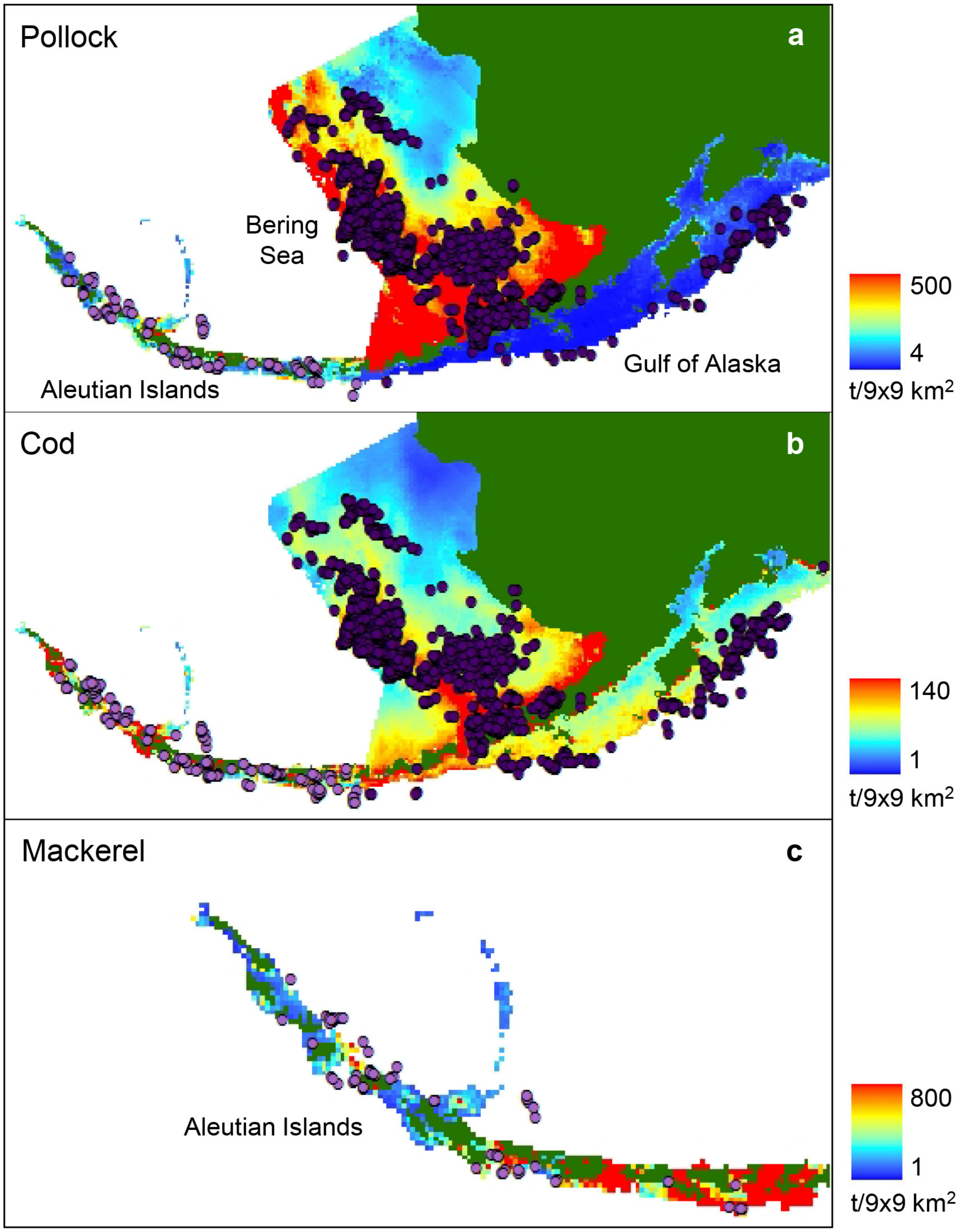

Fig 2. Locations of catches relative to predicted biomass distributions. Biomass distributions (t/9x9 km^2 grid cell) are shown for (a) walleye pollock available in the Aleutian Islands (2000), Bering Sea (2001) and Gulf of Alaska (2001), (b) Pacific cod available in the Aleutian Islands (2002), Bering Sea (2003) and Gulf of Alaska (2003), and (c) Atka mackerel available in the Aleutian Islands (2004) (modified from S1). Locations of catches (dots) shown are from the same years as the corresponding prey distributions (Aleutian Islands: pale purple, Bering Sea and Gulf of Alaska: dark purple).

doi:10.1371/journal.pone.0123786.g002

locations of sea lions from satellite telemetry provided by NMFS and the Alaska Department of Fish and Game (ADFG) to estimate the likelihood of sea lions occurring at different distances from their haulouts and rookeries. A total of 116 sea lions (pups and juveniles) were tracked by satellite from 2000–2005 in the Aleutian Islands and Gulf of Alaska primarily during spring and summer, with a few tracked in the fall of 2001. The tags typically transmitted data for 1–3 months (Brian Fadely, NMML-NMFS, pers. comm.) and yielded 2–523 locations per sea lion (124 ± 44 locations, mean ± s.e., n = 116 sea lions). These data were filtered for quality and estimated to be within 150 to 1,000 m of the true location of the animal. We assumed the tagged animals were representative and that their movements were unaffected by the tags.

To increase the accuracy of our foraging distance assessment, we omitted outliers (> 100 km) and animals located at sea < 30 times from our analysis. We grouped the telemetry records into two age categories of sea lions (<10 months of age and >10 months of age) based on the presumed age at weaning [47–49].

We binned the retained telemetry data into 1 nautical mile intervals from the nearest rookery or haulout shoreline (straight line distance) and calculated the proportion of locations within each 1 nautical mile bin for each age group. We tested for differences between sex and region (Aleutian Islands vs. Gulf of Alaska).

We fitted models to the proportion of individuals at each 1 nautical mile interval by age group, region and sex—and transformed the data where necessary to look for similarities in trends between distance intervals. We used the model which best described the observed distance of sea lions from shore to calculate accessibility.

Combining prey accessibility with available prey biomass

We assumed that prey occurring closer to rookeries were more important to sea lions than prey further away based on sea lions foraging closer to their rookeries in summer than in winter. This implies that prey biomass should be scaled to reflect its accessibility, as a function of distance from the rookeries.

We created an accessibility surface by first calculating the distance from each grid cell to each rookery using the IDRISI Distance function [50]. For each rookery, we then applied the accessibility model to classify the at-sea pixels into accessibility classes. To calculate the likelihood of a sea lion occurring within each grid cell, we divided the accessibility value of each grid cell by the sum of all accessibility values within the foraging area of each rookery, defined as all pixels with non-zero accessibility. The final accessibility values for each rookery's foraging area thus summed to 1.

We next multiplied the predicted prey biomass by each rookery's accessibility surface, thereby assigning higher importance to prey resources that were closer to the rookery. We then summed these estimated accessible biomasses of pollock, cod and mackerel for each rookery from 2000–2004 (2000, 2002 and 2004 in the Aleutian Islands; 2001 and 2003 in the Gulf of Alaska).

We also compared the total biomass of pollock, cod and mackerel within 10, 20 and 50 km of each rookery with the predicted accessible biomass from 2000–2004 (2000, 2002 and 2004 in the Aleutian Islands; 2001 and 2003 in the Gulf of Alaska). These distances were based on estimated foraging distances of sea lion females and juveniles in summer [28–32,51,52]. Thus, we considered the possibility that accessibility of prey declined with distance from shore, as well as the possibility that all prey within 10, 20 and 50 km of shore was available and equally accessible to the sea lions (i.e., accessibility value of each grid cell within the selected ringed distances was 1). Beyond these selected distances, the likelihood of sea lion foraging was assumed to be 0.

Statistical analyses

Spatial autocorrelation. Rookeries that are close to each other tend to have similar population sizes and trends over time [41,44,45]. They also share some portion of their accessible prey biomasses. To remove this spatial autocorrelation from the statistical analysis we grouped rookeries that were within 50 km of each other into clusters, thus assuming that sea lions in these rookery clusters shared a common prey base. We examined semivariograms of the differences in rates of sea lion population change from 2000–2008 and the distances between rookeries to confirm that our 50 km grouping was sufficient for reducing any spatial autocorrelation. We then used a weighted average to calculate the proportion of shared prey biomass accessible to the sea lions at each rookery within a cluster:

$$\frac{N_A fish_A}{N_A + N_B} + \frac{N_B fish_B}{N_A + N_B} \quad (1)$$

for a cluster consisting of rookeries A and B, where N_A and N_B are the population size estimates of pups or non-pups at rookeries A and B respectively, and $fish_A$ and $fish_B$ are the biomasses of pollock, cod or mackerel accessible to rookeries A and B respectively.

Relationship between sea lion population change and prey abundance. We used linear mixed-effects (LME) (Fig 3a and 3c) models to test whether the relationship between the annual rate of sea lion population change, the biomass of pollock, cod or mackerel accessible to each rookery (or rookery cluster), and the region were significant across all rookeries considered. LME models allowed us to characterise the variation within rookeries relative to the mean of all rookeries while considering the correlation between repeated measurements within the same rookery. We included prey biomass and region as fixed effects. Repeated measurements on each rookery across years were treated as a random effect for all models. The models fitted were of the general form:

$$r_{i,j,k} = \beta_0 + \beta_{region} region_k + \beta_{fish} fish_{i,j,k} + \beta_{fish:region} fish_{i,j,k} : region_k + b_{j,k} + \varepsilon_{i,j,k} \quad (2)$$

where $r_{i,j,k}$ is the annual rate of sea lion population change (pups or non-pups) in the ith year at the jth rookery (or rookery cluster) in the kth region,

β_0 is the intercept,

β_{region} is the coefficient for regional effects,

$region_k$ is the region-specific intercept used to test for differences between regions,

β_{fish} is the coefficient for accessible prey biomass (pollock, cod or mackerel),

$fish_{i,j,k}$ is the biomass of pollock, cod or mackerel accessible in the ith year to the jth rookery (or rookery cluster) in the kth region,

$\beta_{fish:region}$ is the coefficient for the interaction between accessible prey biomass and region,

$b_{j,k}$ is the random effect associated with the jth rookery (or rookery cluster) in the kth region, assumed to be independent from the other rookeries,

":" represents the interaction between fish biomass and region,

and $\varepsilon_{i,j,k}$ is the independent, homogenously distributed within-rookery error associated with the jth rookery (or rookery cluster) in the kth region in the ith year, assumed to be independent of the random effects.

The annual rate of sea lion population change $r_{i,j,k}$ was calculated as

$$\ln\left(\frac{N_{i+1,j,k}}{N_{i,j,k}}\right) \quad (3)$$

where $N_{i,j,k}$ is the population size estimate of non-pups or pups (which do not subsist on fish,

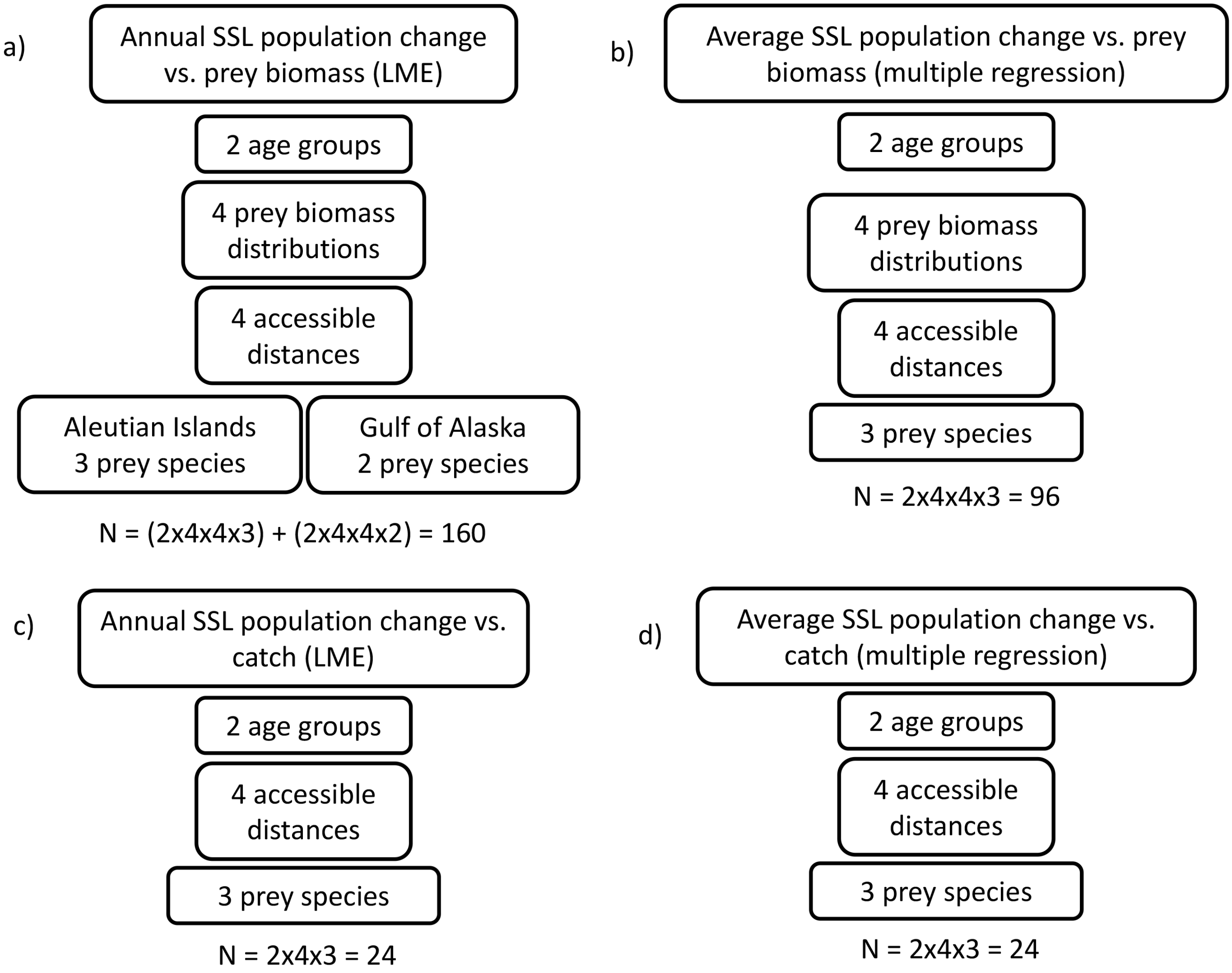

Fig 3. Statistical model types and derivations used to test the relationship between sea lion population change and prey abundance (a and b); and between sea lion population change and fisheries catch (c and d). Multiple regression and linear mixed-effects models (LME) were used to test for relationships between different age groups of Steller sea lions (SSL), prey biomass distributions, catch, accessible distances and regions. Derivation of the number omodels (N) analysed with each different combination is shown.

doi:10.1371/journal.pone.0123786.g003

but may be affected by reduced prey availability to their mothers) in the ith year at the jth rookery (or rookery cluster) in the kth region. For rookery clusters, we calculated annual rate of sea lion population change as

$$\ln\left(\frac{N_{A,i+1,j,k} + N_{B,i+1,j,k}}{N_{A,i,j,k} + N_{B,i,j,k}}\right) \tag{4}$$

for a cluster consisting of rookeries A and B, where $N_{A,i,j,k}$ and $N_{B,i,j,k}$ are the population size estimates of pups or non-pups at rookeries A and B respectively in the ith year at the jth rookery cluster in the kth region. Annual rates of sea lion population change were log transformed to normalise the data and homogenise the distribution of the variances (within-rookery errors).

Region was included as a fixed effect in the models because much of the variability in Steller sea lion population trends has been attributed to regional differences in oceanography [41,42,45] and diet [53]. As variability in population change was greater among rookeries in the western Aleutian Islands than in the eastern Aleutian Islands (heterogeneity of variances), we chose a model that incorporated regional variances as a measure of within-rookery errors. We fitted separate models for (1) Aleutian Island and Gulf of Alaska rookeries, (2) pup and non-pup population changes, (3) each of the three prey species, (4) each of the four predicted distributions of prey, and (5) for each of the chosen distances and the accessibility model. This generated 160 models (Fig 3a), all of which were fit using the maximum likelihood method. The intercept was allowed to vary for each rookery or rookery cluster during model optimisation.

For each of the 160 models, the best model structure was determined using likelihood ratio tests (LRTs) and Akaike's information criterion (AIC). An Analysis of Variance (ANOVA) performed on two nested models (the simpler model nested within the more complex model) produced a LRT that compared the likelihoods of the models in explaining the relationship between the independent and dependent variables. AIC was calculated from the number of parameters and the likelihood function of the model.

We investigated more general or long-term relationships between sea lion population trends and accessible prey biomasses by comparing the average annual rate of sea lion population change (pups or non-pups) from 2000–2008 with the average biomass of pollock, cod or mackerel accessible to each rookery (or rookery cluster) across all available years using a multiple regression model (Fig 3b and 3d):

$$\lambda_{j,k} = \beta_0 + \beta_{region} region_k + \beta_{fish} fish_{j,k} + \beta_{fish:region} fish_{j,k} : region_k + \varepsilon_{j,k} \quad (5)$$

where $\lambda_{j,k}$ is the average annual rate of sea lion population change (pups or non-pups) from 2000–2008, calculated from linear regressions of log transformed population estimates from Winship and Trites (2006) and Battaile and Trites (supplementary data will be provided if manuscript is accepted), at the jth rookery (or rookery cluster; average annual rates of change at rookery clusters were calculated from the total estimated population size of each cluster's constituent rookeries) in the kth region (eastern or western Aleutian Islands; or eastern or western Gulf of Alaska),

β_0 is the regression intercept,

β_{region} is the regression coefficient for regional effects,

$region_k$ is the region specific intercept used to test for differences between regions,

β_{fish} is the regression coefficient for average accessible prey biomass (pollock, cod or mackerel),

$fish_{j,k}$ is the average biomass of pollock, cod or mackerel accessible across all years (2000, 2002 and 2004 in the Aleutian Islands; 2001 and 2003 in the Gulf of Alaska) to the jth rookery (or rookery cluster) in the kth region,

$\beta_{fish:region}$ is the regression coefficient for the interaction between average accessible prey biomass and region,

":" represents the interaction between fish biomass and region,

and $\varepsilon_{j,k}$ is the independent, homogenously distributed error associated with the jth rookery (or rookery cluster) in the kth region.

Separate models were fitted for (1) pup and non-pup population change, (2) each of the three prey species, (3) each of the four types of prey distributions, and (4) each of the chosen distances and the accessibility model (total of 96 models, Fig 3b). Again, the simplest model with the fewest number of parameters which could explain most of the variation in the average

annual rate of sea lion population change was determined using LRTs and AIC. We used F tests to determine the significance of the regression coefficients; and reported all results as mean ± standard error; significance tests were conducted at the α = 0.05 level.

Relationship between fisheries catch and sea lion population change. We tested for a relationship between the annual biomass of pollock, cod and mackerel caught by fisheries, and the annual rates of sea lion population change because catch distributions outside the Steller sea lion's breeding season may be important as substantial fishing for pollock, cod and mackerel occurs in fall and winter. Analysing catch data also gives an indication of prey availability in high biomass areas where fishing and probably sea lion foraging are most intense.

We summed the biomass of pollock, cod and mackerel caught by fisheries within 10, 20, 50 and 100 km of each rookery for each rookery from 2000–2004 (e.g., Fig 4). We included the 100 km distance because Steller sea lions travel further in winter [28–32,51,52]. We used the same rookery clusters and associated formulas to minimise spatial autocorrelation. Fisheries catch was then compared to sea lion population change using LME and multiple regression models similar to Models 2 and 5 above, except that we used the biomass of pollock, cod or mackerel caught annually by fisheries in place of accessible prey biomass in the model equations. We analysed catch relationships with sea lion population change in all four regions together as catch data were available in all regions every year from 2000–2004. Separate models were fitted for (1) pup and non-pup population change, (2) each of the three prey species and (3) selected distances resulting in 24 yearly LME (Fig 3c) and 24 long-term average multiple regression models (Fig 3d). The biomass of Atka mackerel caught was only considered for the Aleutian Island sea lion rookeries because there has not been a directed fishery for mackerel in the Gulf of Alaska since 1996.

Results

Sea lion population trends and fishery catches

The decline of the western stock of Steller sea lions from 2000–2008 was driven by declines at several rookeries in the Aleutian Islands (Fig 5). Mean annual rates of change for all Aleutian Island rookeries were -1.7% for non-pups and -1.9% for pups. In contrast, sea lions in the Gulf of Alaska increased at an overall annual rate of 2.4% for non-pups and 2.8% for pups. Rookery sizes tended to be smaller in the Aleutian Islands than in the Gulf of Alaska, averaging 215 pups and 366 non-pups in the Aleutians, and 326 pups and 588 non-pups in the Gulf.

Examination of the semivariograms of the differences in average annual rates of sea lion population change from 2000–2008 and the distances between rookeries confirmed a gradual increase in similarity in population trends as the distances between rookeries decreased. Based on the semivariograms, we concluded that grouping rookeries within 50 km of each other into clusters was sufficient to reduce the potential for spatial autocorrelation between rookeries. This grouping resulted in 16 single rookeries and 8 rookery clusters (Agattu Gillon Point and Agattu Cape Sabak; Kiska Cape St Stephen and Kiska Lief Cove; Ayugadak and Amchitka Column Rock; Ulak Hasgox Point, Tag and Gramp Rock; Adugak and Ogchul; Akutan Cape Morgan and Akun Billings Head; Clubbing Rocks North and Pinnacle Rock; and Chernabura and Atkins) (Fig 5). Accessible prey biomasses and rates of population change were calculated by rookery or rookery clusters.

Commercial catch levels differed greatly between species, rookeries and distances from the rookeries. Annual catches of mackerel within 100 km of the rookeries were highest in the western Aleutians, whereas within 50 km, catches were highest in the central Aleutians (Fig 6a). Annual catches of pollock and cod within 50 and 100 km of the rookeries were highest in the Bering Sea (Fig 6b and 6c). Overall, there was relatively little catch within 20 km of the rookeries

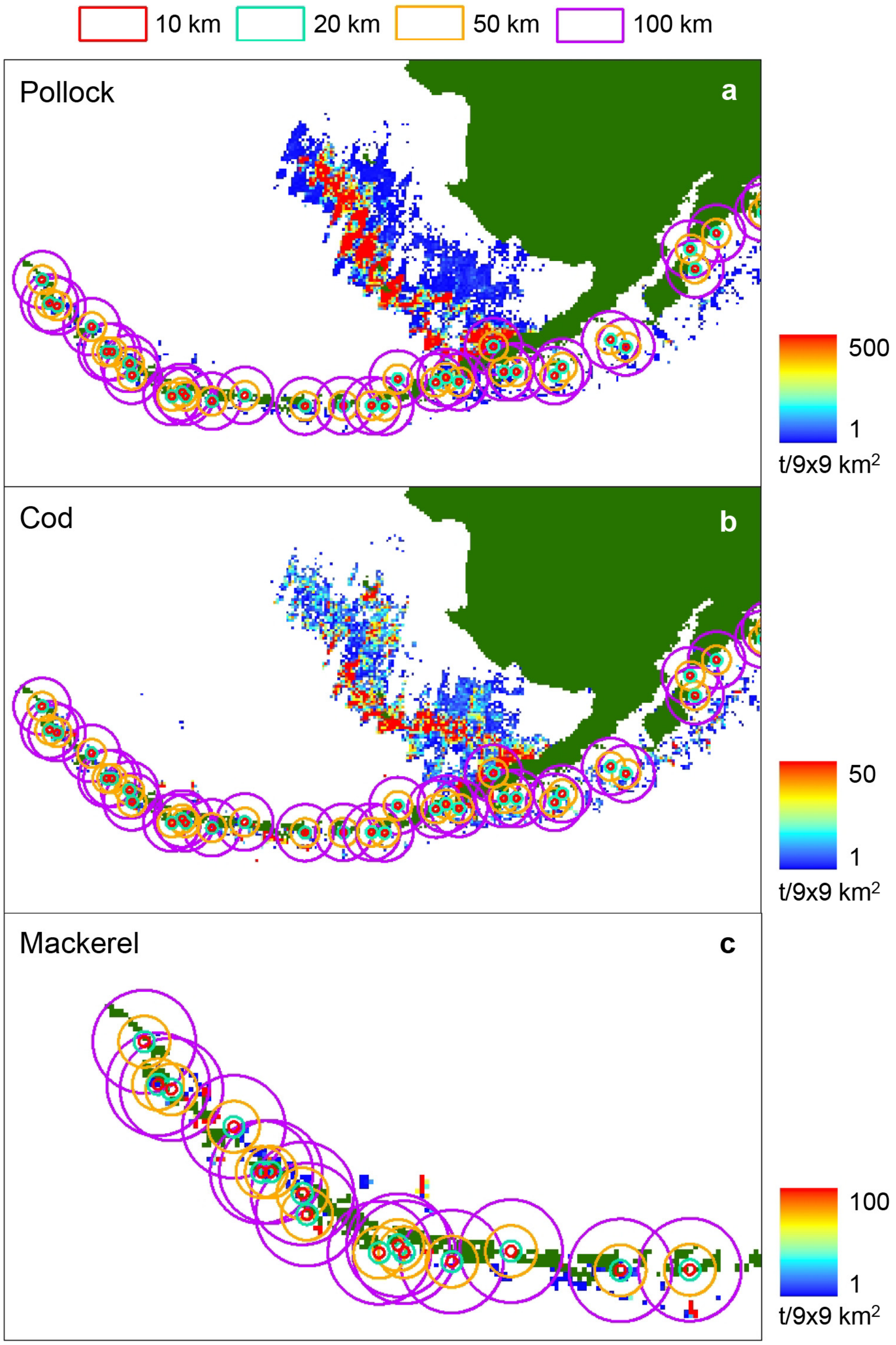

Fig 4. Annual catch in t/9x9 km^2 of (a) walleye pollock (2003), (b) Pacific cod (2002) and (c) Atka mackerel (2004). Total amounts removed within 10, 20, 50 and 100 km of each rookery (red, cyan, orange and purple rings respectively) were calculated by summing the total biomass of catches within each of the respective rings.

doi:10.1371/journal.pone.0123786.g004

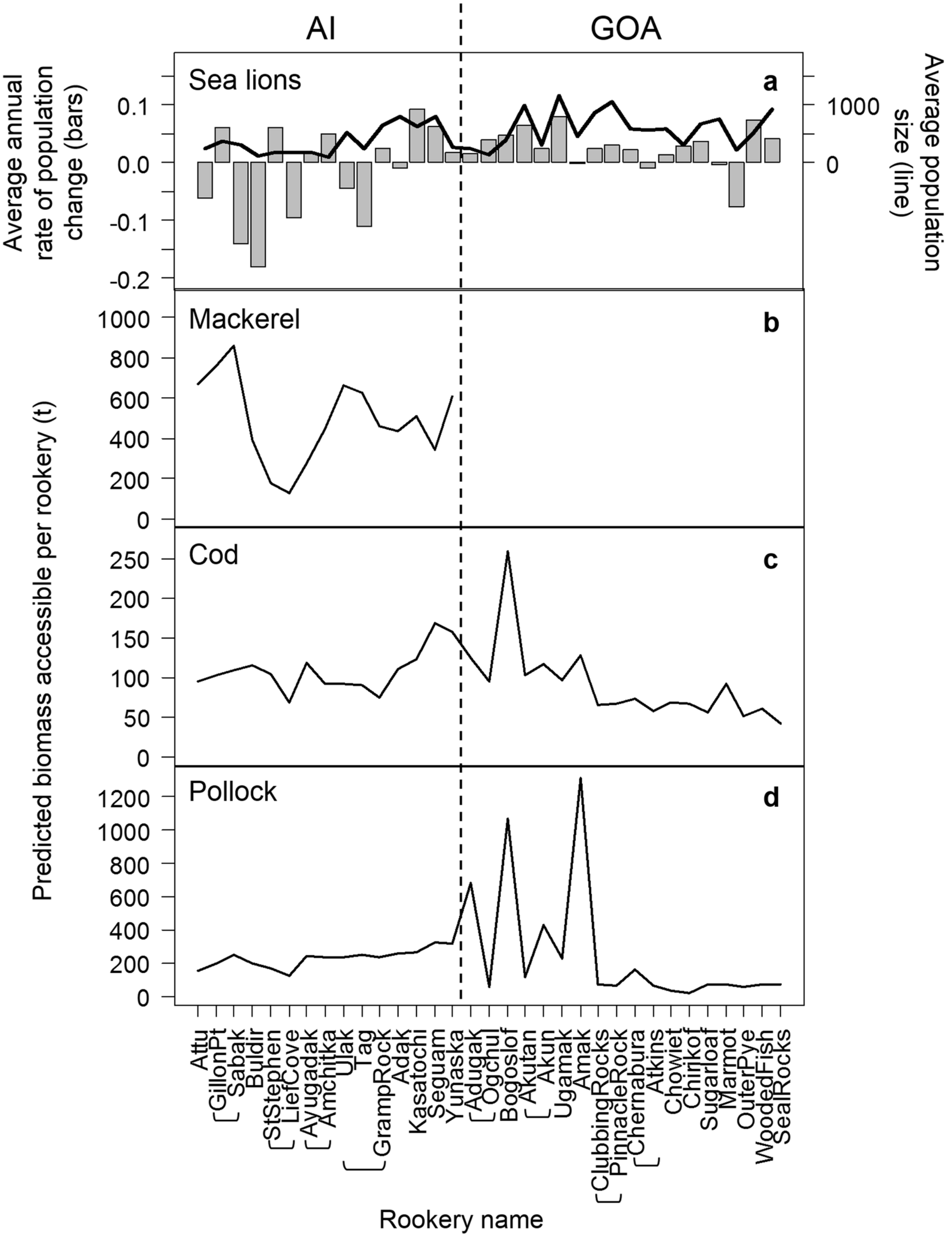

Fig 5. Sea lion population change and the biomass of prey accessible to sea lions. The average numbers of Steller sea lions (age 1+ y), annual rate of sea lion population change and predicted biomass of groundfish accessible (calculated according to our accessibility model) to sea lions at each rookery or rookery cluster (shown with brackets): (a) Average non-pup population change and population size from 2000–2008, (b) average biomass of Atka mackerel accessible, (c) average biomass of Pacific cod accessible; and (d) average biomass of walleye pollock accessible. Biomasses averages are for 2000/2002/2004 in the Aleutian Islands (AI) and 2001/2003 in the Gulf of Alaska (GOA). Mackerel surveys are not conducted in the Bering Sea and Gulf of Alaska as the species' distribution is limited in those regions.

doi:10.1371/journal.pone.0123786.g005

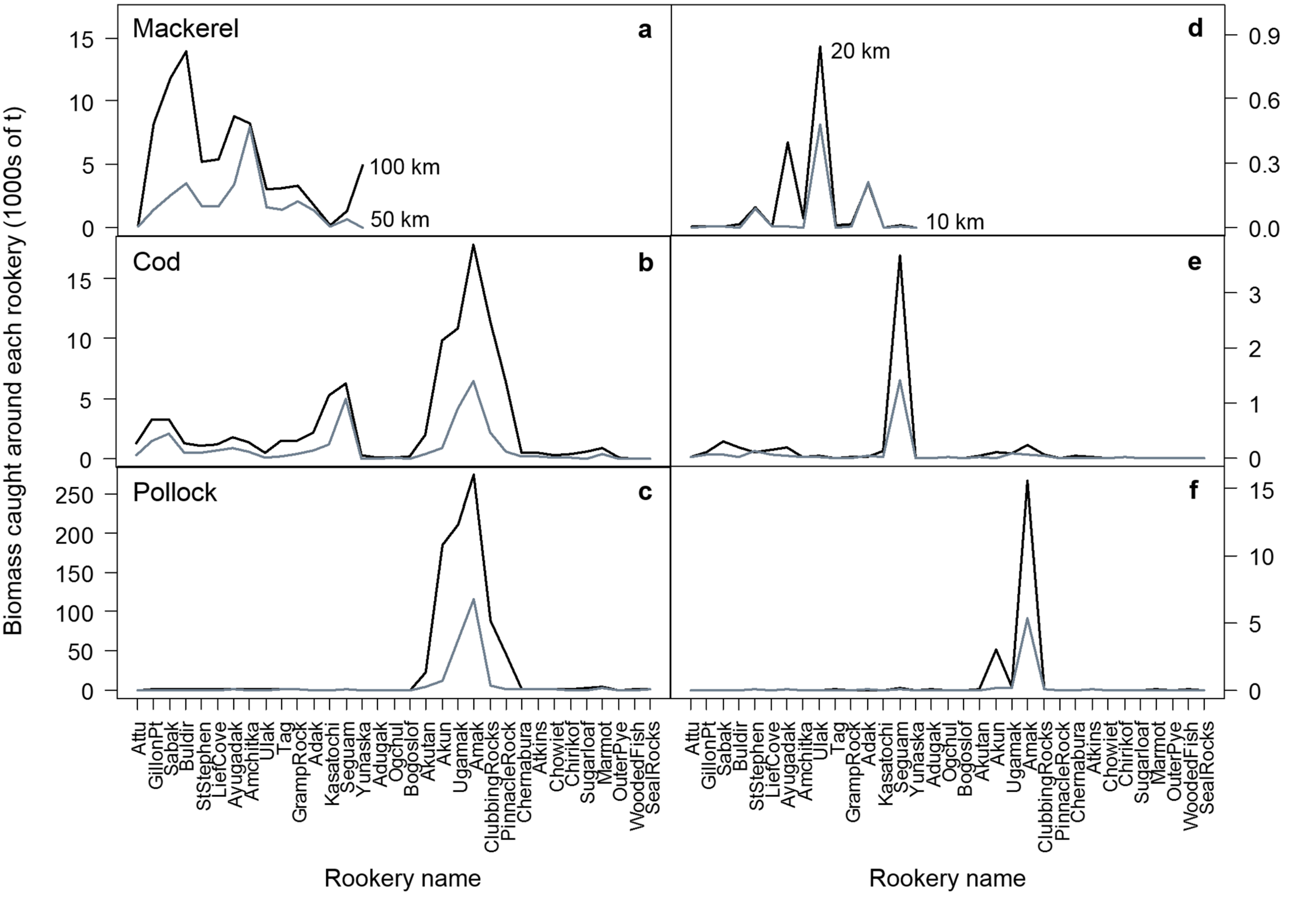

Fig 6. Annual biomass of pollock, cod and mackerel caught by fisheries. Average annual biomass (1000s of tons) of Atka mackerel (a,d), Pacific cod (b, e) and walleye pollock (c,f) commercially caught within 10 and 20 (d, e, f) and 50 and 100 (a, b, c) km of the rookeries from 2000–2004. There has been no directed fishery for mackerel in the Bering Sea and Gulf of Alaska since 1996.

doi:10.1371/journal.pone.0123786.g006

(Fig 6d–6f). Region was the only significant factor influencing sea lion population trends ($F_{3,20}$ = 6.74, P = 0.0025), which were lowest (more negative) in the western Aleutian Islands but increased (becoming more positive) toward the Gulf of Alaska. No significant relationships were found in the 48 models fit to catch and sea lion population change (Fig 3c and 3d).

Distributions of sea lions and prey

The sample of screened telemetry data (n = 86) showed that accessibility declined exponentially for sea lions older than 10 months as a function of distance from shore. We log-transformed the data to look for similarities in trends between distance intervals and detected a change point around 17 nautical miles from shore. We fit an exponential decay function to the average proportions of locations as far as 17 nautical miles and found that this model gave reasonable predictions beyond 17 nautical miles when compared to the original data points (Fig 7a). Attempts to fit an additional model to describe accessibility beyond 17 nautical miles did not improve the overall model predictions. The extremely low proportions of locations beyond 17

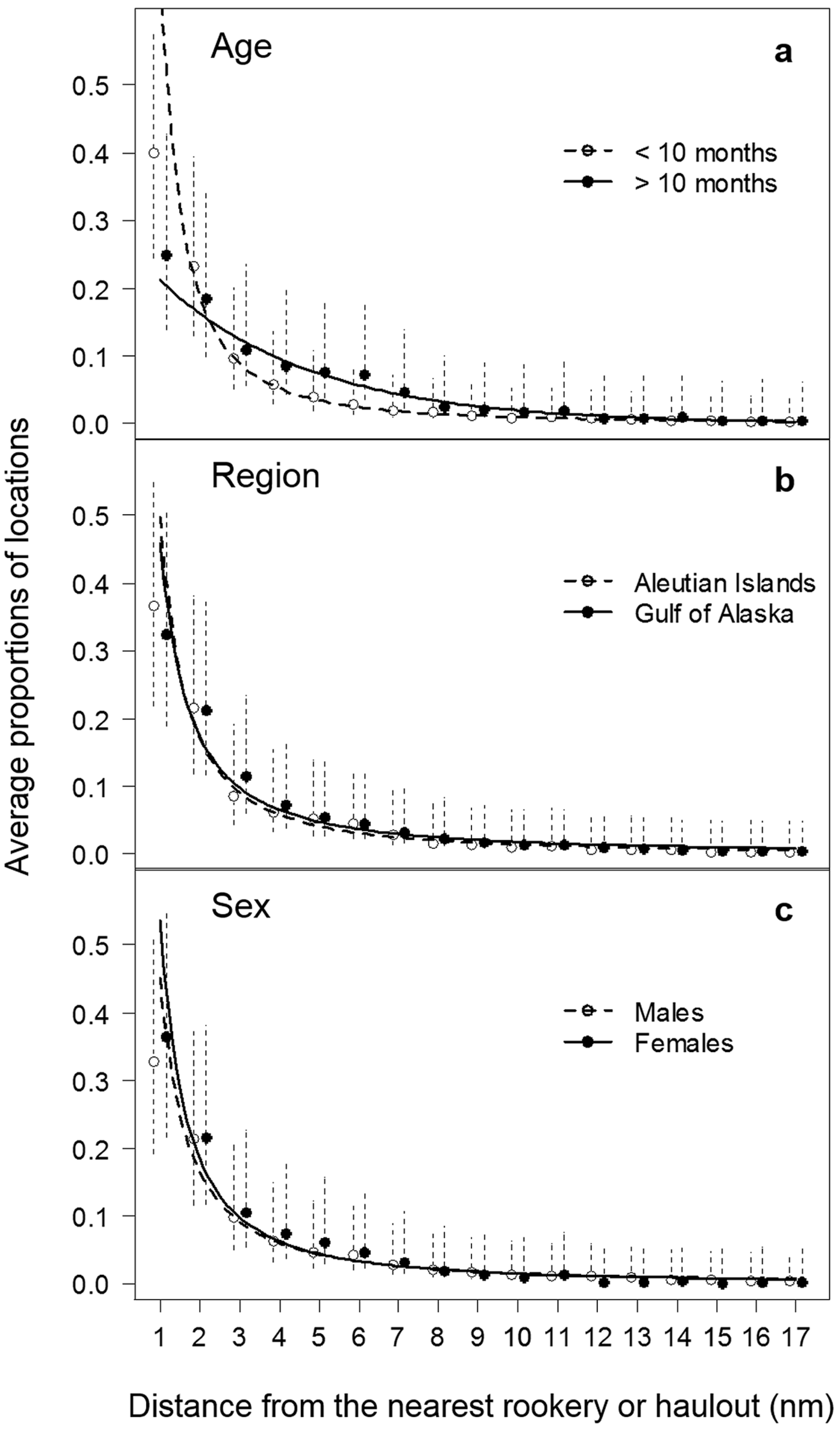

Fig 7. Average proportion of locations in each distance interval (in 1 nautical mile increments). Proportions are shown for (a) Steller sea lions older (n = 33) ($y_{>10} = 0.2756e^{-0.2639x}$) and younger (n = 56) ($y_{<10}$ = 0.6757x-1.8506) than 10 months of age, (b) sea lions from the Aleutian Islands (n = 41) (yAI = 0.4964x-1.5478) and Gulf of Alaska (n = 48) (yGOA = 0.4591x-1.4068), and (c) male (n = 51) (ymale = 0.4512x-1.4515) and female (n = 38) (yfemale = 0.5367x-1) sea lions. Proportions are shown as mean ± s.e. Telemetry data were provided by Brian Fadely, NMFS.

doi:10.1371/journal.pone.0123786.g007

nautical miles (<0.003) indicated that adult female and juvenile sea lions rarely forage beyond 17 nautical miles in summer.

For sea lions younger than 10 months, we found their at-sea distribution was best described by a power function (Fig 7a). These younger sea lions spent smaller proportions of time at greater distances from land compared to sea lions older than 10 months (Fig 7a). Power functions also provided the best fit to the data for comparisons between males and females, and between regions (Aleutian Islands versus Gulf of Alaska). The at-sea distributions of sea lions were similar between regions (Fig 7b) and showed no differences between males and females (Fig 7c). We therefore used the data from sea lions older than 10 months (which were more likely to be representative of foraging animals; n = 33) to describe accessibility to prey. We extended the model predictions up to 99 km (~53.46 nautical miles, 1 nautical mile = 1.852 km) in the accessibility model grids (i.e., eleven 9 x 9 km^2 grid cells) to reflect the possibility (though extremely slight) of sea lions foraging far away from their rookeries.

By applying the accessibility model to the predicted available biomass we significantly reduced the prey available to sea lions (i.e., biomasses were in the hundreds of tons using the accessibility model (Fig 5b–5d), and in the thousands of tons without the accessibility model (Fig 8). In addition to reducing the total prey biomass accessible at each rookery, the accessibility model also reduced the accessible biomasses among rookeries. In particular, rookeries with exceptionally high predicted available biomass within 20 and 50 km (Fig 8) had only moderately high accessible biomasses (Fig 5b–5d) because most high biomass areas were located further away from the rookeries.

Of the 256 sea lion population change-prey biomass models fitted, we found 3 significant relationships. The biomass of pollock accessible under Scenario 3 was positively associated with non-pup population change in the Aleutian Islands ($F_{1,18} = 4.57$, $P = 0.046$) (Fig 9). This included a significant interaction between biomass and region ($F_{1,18} = 8.67$, $P = 0.0087$), with western Aleutian Island rookeries showing a greater response to pollock biomass (Fig 9). In other words, population increases were significantly associated with more pollock in the Aleutian Islands, particularly in the western Aleutians.

We found positive relationships between the biomass of cod accessible to sea lions (both unreduced and in Scenario 1) and the annual rate of non-pup population change in the Gulf of Alaska ($F_{1,13} = 4.85$, $P = 0.046$). Removing a single outlier with more than 400 t of cod notably strengthened the relationship (reduced and unreduced: $F_{1,12} = 16.11$, $P = 0.0017$) (Fig 10b and 10d). The results with and without fishery removals were very similar due to the small amount of cod removed within the model extents in June and July (Fig 10b and 10d).

None of the models for Atka mackerel, nor for the other two prey species within 10, 20 or 50 km, were significant. Neither were any models that tested for effects on pups. None of the 96 multiple regression models used to examine the long-term relationships between sea lion population trends and accessible prey biomasses were significant.

Discussion

Interspecific competition is defined as a reduction in the fecundity, growth or survivorship in the individuals of one species as a result of resource exploitation by another species [54]. Determining whether competition exists between fisheries and sea lions requires demonstrating that the species being caught and eaten are limited. We sought to improve on past studies that attempted to assess potential competition between sea lions and commercial fisheries by considering the distribution of prey accessible to sea lions, and not just the prey removed by fisheries or fishing effort. We therefore compared sea lion population trends with spatially-explicit distributions of prey to assess whether the observed rates of sea lion declines were related to the

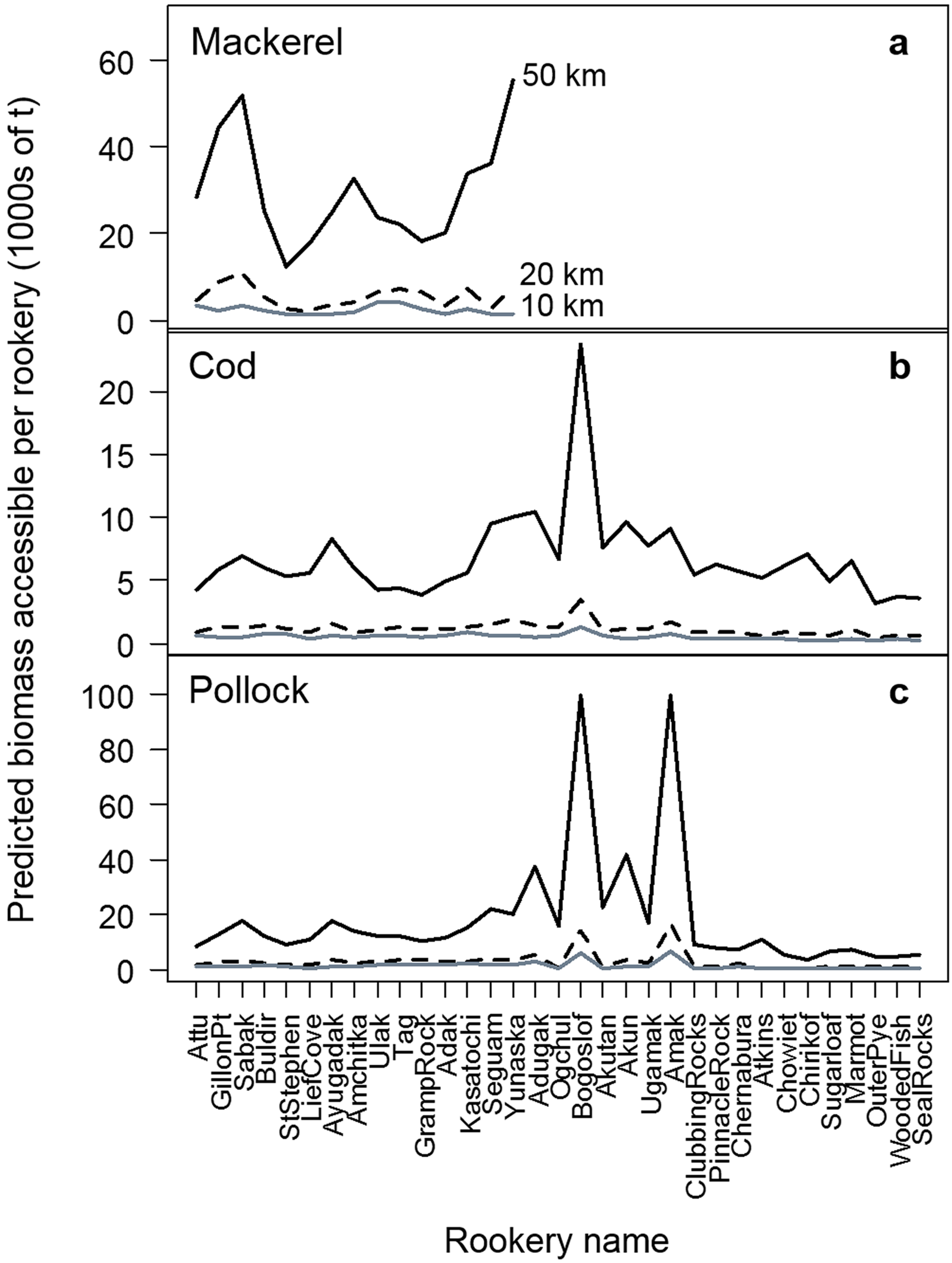

Fig 8. Predicted prey biomass accessible to sea lions. Predicted biomass (1000s of tons in the absence of fishing) of (a) Atka mackerel, (b) Pacific cod and (c) walleye pollock accessible to Steller sea lions within 10, 20 and 50 km of the rookeries. Biomasses are averages for 2000/2002/2004 in the Aleutian Islands (AI) and 2001/2003 in the Gulf of Alaska (GOA). No data were available for Atka mackerel in the Bering Sea and Gulf of Alaska due to the small amounts predicted to occur in those regions.

doi:10.1371/journal.pone.0123786.g008

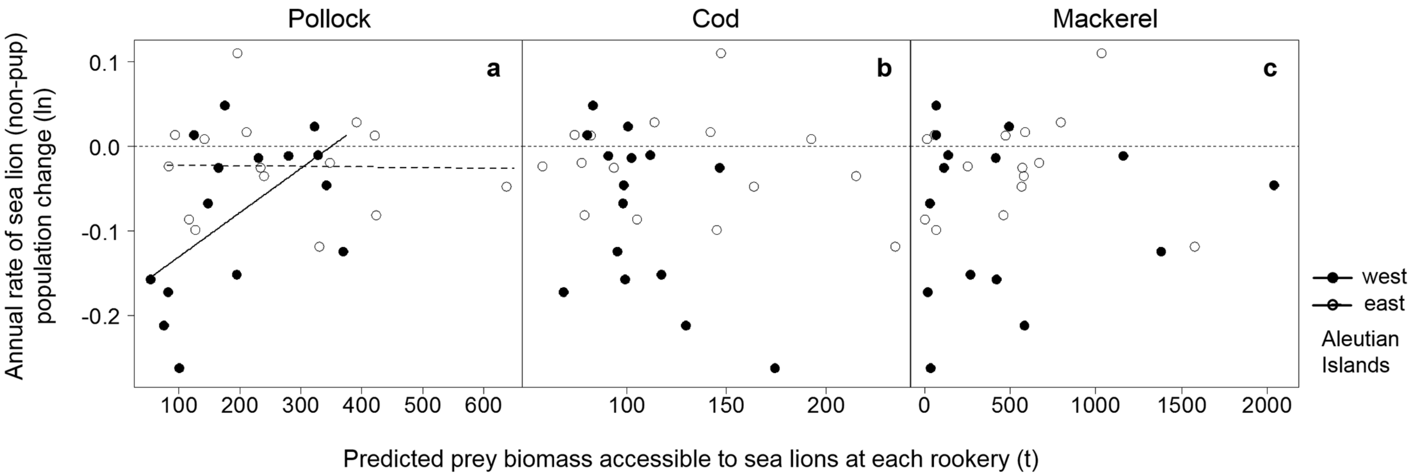

Fig 9. Relationships between accessible prey biomass and sea lion population change in the Aleutian Islands. The relationships between predicted prey biomass accessible to Steller sea lions (a, b, c) using the reduced prey biomass (Scenario 3) and the annual rate of non-pup population change in the Aleutian Islands were significant for walleye pollock only (a), with western Aleutian rookeries (west, from rookeries 1–8, see Fig 1) showing a greater change with pollock biomass than eastern Aleutian rookeries (east, from rookeries 9–15, see Fig 1).

doi:10.1371/journal.pone.0123786.g009

availability and accessibility of prey, or the amount of commercial catch. The analysis allowed us to detect the potential for localised depletion of prey to have occurred around individual rookeries. Models which are capable of identifying the spatial and temporal distributions of prey are important for facilitating the planning and optimisation of management actions [55,56]. For example, Margalida & Colomer [57] were able to use temporally and spatially dynamic models to predict the effects of prey removal on vulture populations. Including sea lion accessibility in our models gave a more realistic representation of the prey available to sea lions by taking into account their foraging behaviour [58,59].

Importance of pollock and cod in the diet of sea lions

Our study took the approach of creating localised estimates of prey biomass available while accounting for the accessibility of sea lion prey and their foraging behaviour. We also examined the potential impacts of fisheries removals on prey availability by predicting four possible scenarios of fisheries removals and fish redistribution following fishing. However, we only found three statistically significant relationships between prey abundance and sea lion population change out of a total of 256 models comparing the accessible biomass of pollock, cod and mackerel to sea lion population trends. All three relationships suggested sea lion populations increased (became less negative) with increasing accessibility of prey during summer. While these relationships may be spurious given the number of models we fitted, they do make biological sense.

Pollock move to shallower waters for spawning and feeding in summer, making them more accessible to sea lions [6,60,61]. In addition, the lipid content of pollock peaks in summer [62], providing sea lions with a relatively more nutritious prey source.

Pacific cod has been one of the four most important prey items of Steller sea lions in terms of frequency of occurrence averaged over years, sites and seasons, and has been especially important in winter [53,63]. Pitcher [64] also found that Pacific cod was an important winter prey item in the Gulf of Alaska, as did Calkins [65] in the Bering Sea. The increase in sea lion numbers with cod biomass in summer is somewhat surprising however, as cod have a relatively low summer energy density (post-spawning) compared to winter (pre-spawning) [66].

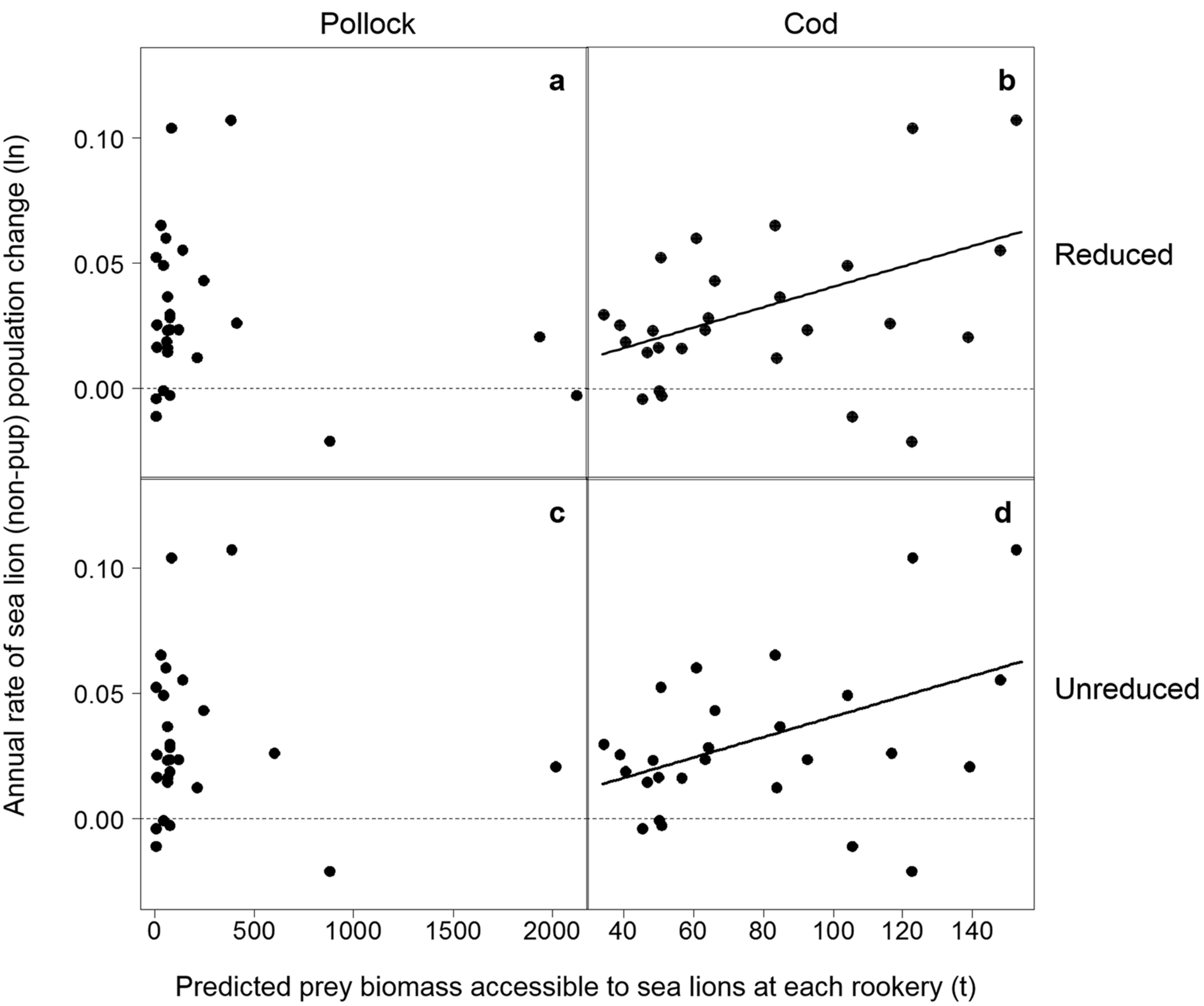

Fig 10. Relationships between accessible prey biomass and sea lion population change in the Gulf of Alaska. The relationships between predicted prey biomass of walleye pollock (a, c) and Pacific cod (b, d) accessible to sea lions using the reduced (Scenario 1; a, b) and unreduced biomass distributions (c, d), and the annual rate of non-pup population change in the Gulf of Alaska were significantly positive for Pacific cod only. The trends with and without fishery removals accounted for were very similar due to the small amount of cod removed within the accessibility model extents of the rookeries in June and July.

doi:10.1371/journal.pone.0123786.g010

Moreover, most cod move to the outer shelf in summer in accordance with their annual migration cycle [67]. Adult cod are known to prey on juvenile pollock [68], and cod are often caught together with pollock [69]—so the few cod which remain in shallow waters may form an important food source in addition to pollock.

Regional differences in sea lion population trends

The region in which the rookery was located influenced the relationship between pollock biomass and sea lion population trends, with western Aleutian Island rookeries showing a greater change than eastern Aleutian Island rookeries. Population change rates tended to be greater and

more negative among the western Aleutian Island rookeries compared to the eastern Aleutian Island rookeries, as well as among the Aleutian Island rookeries compared to the Gulf of Alaska rookeries. Our results support the emerging understanding that regional oceanographic differences can influence the distribution and abundance of prey available to sea lions [42,43,70,71].

Sea lion accessibility

Steller sea lions are central place foragers [72], regularly resting on land between foraging trips [28,30,47,73,74]. Their rookeries and haulouts are likely chosen, in part, for their proximity to prey resources [75]. We thus expected sea lions to concentrate most of their foraging closest to their rookeries and haulouts. We found evidence to support this prediction, with the number and proportion of locations for each sea lion decreasing exponentially with increasing distance from the nearest rookery or haulout. We felt that it was reasonable to assume that our accessibility model (which was based on satellite telemetry locations from juvenile sea lions) was a reasonable proxy for adults as well because juveniles forage at similar distances from rookeries and haulouts as adults [28–32,51,52].

The accessibility of foraging areas from a central place has been described for birds and mammals using linear equations and normal density functions. For example, the likelihood of seabirds foraging near nesting sites has been assumed to decrease linearly with distance from land [76], while Gregr and Trites [58] modelled the accessibility of a foraging area from a Steller sea lion rookery or haulout using the positive half of a normal density function. However, both these descriptions contrast with our telemetry-based model that shows accessibility decreases exponentially with increasing distance from land. The telemetry data we used suggest there is no distance within which accessibility by sea lions is more or less equal as suggested by the initial plateau of a normal curve. Our results suggest that foraging areas closest to the rookeries may be many orders of magnitude more important than foraging areas further away, at least during the spring and summer months. While sea lions are known to forage further from shore in winter [28], the exponential relationship between distance and accessibility may well hold during winter as well, assuming the animals are returning to their starting locations to haul out and rest.

Foraging patterns observed for other species confirm that most individuals make short foraging trips, concentrated near central places. For example, the movements of grey seal *Halichoerus grypus* from one location to the next tend to be short (~6 km) and decrease linearly in frequency with increasing movement length [77]. Similarly, the proportion of flights made by wandering albatrosses *Diomedea exulans* decreased exponentially with increasing flight duration [78,79], as did the frequency of feeding behaviour in deer with increasing foraging time (time spent searching for food) [80]. Both studies assumed that time spent travelling between food items or patches was related to the distance travelled. Such observations suggest that accessibility of prey may decrease exponentially with increasing distance from the central place for other species as well.

Are sea lions prey limited?

Only 3 of the 304 regressions we ran comparing the accessible prey biomass and catch of pollock, cod and mackerel to sea lion population change were statistically significant. It is therefore unlikely that the availability of pollock, cod or mackerel was limiting sea lion populations in the 2000s. Any changes in sea lion numbers from year to year in response to changes in pollock, cod or Atka mackerel availability were probably temporary, as we failed to find any significant long-term (i.e., 9 years) relationships between sea lion population trends and accessible prey biomass or catch during our study period. It seems that pollock, cod and mackerel

biomass was high enough, relative to the number of sea lions, that there was no shortage of these three prey species. If sea lions were prey limited, it would most likely be the result of species besides pollock, cod and mackerel.

Steller sea lions are opportunistic, generalist predators and take advantage of prey with strong, predictable, nearshore migratory movements [31,43,53,81]. They appear to consume forage fishes and salmon almost exclusively during their summer spawning season, while other fishes and cephalopods were eaten more frequently in spring and fall [53,63,64]. For example, in southeast Alaska, the median percent biomass contribution of forage fishes in the diet of Steller sea lions rose from 13.5% in winter to 21.9% in summer. The median percent biomass contribution of gadids (mainly pollock and cod) on the other hand, fell from 49.1% in winter to 27.3% in summer [82]. When forage fishes such as herring *Clupea harengus*, sand lance *Ammodytes hexapterus* and capelin *Mallotus villosus* are aggregated nearshore, they are likely more energetically rewarding than groundfish because they are are higher in energy density and lipid content, and are easier to catch than groundfish [83]. Forage fishes and salmon are important components of the diet of the western stock of Steller sea lions, forming about 9.8% and 18.8% respectively of their diet by percentage biomass versus 34.2% for gadids and 23.2% for hexagrammids (mainly Atka mackerel) [84].

Do fisheries compete with sea lions for prey?

Of the three prey species we considered, the only statistically significant relationships with changes in sea lion numbers occurred for cod and pollock (accessible biomass). Pollock biomass and sea lion population change were positively related for just one of the prey biomass distributions considered (i.e., reduced pollock biomass distribution under Scenario 3). There was also a positive relationship between cod biomass and sea lion population trends, which did not change regardless of whether we accounted for fishery removals (due to the small amount of cod removed within the accessibility model extents of the rookeries in June and July). The similarities between the four prey distribution scenarios further suggest there was little effect of fishing on the prey available to sea lions. In other words, we could not detect an effect on sea lion numbers when we assumed fishing did not reduce prey biomass (unreduced distributions), or under any of our prey removal scenarios.

The prey biomass and catch distributions showed that areas with relatively higher biomass tended to be further away from the rookeries around the shelf break where fishing was heaviest, but sea lion accessibility was lower. Further reductions of pollock and cod fishing within approximately 99 km (the extent of the accessibility model around each rookery) during summer are therefore unlikely to produce any significant changes in sea lion numbers. Moreover, we did not find any significant relationships between amounts of groundfish caught and sea lion population change to support the hypothesis that fisheries negatively affected sea lions during our study period.

Differences in the timing and magnitude of regional sea lion population trajectories in the 1970s, 1980s, and 1990s suggest that the overall decline of the western stock may not have been caused by a single factor, but rather by the cumulative effect of multiple factors that had different relative spatial and temporal magnitudes [45,85–87]. Ecosystem models of the central and western Aleutians, and southeast Alaska suggest that killer whale predation, ocean productivity, fisheries and competition with other species may have all contributed to the trends observed in sea lion numbers in both ecosystems [26]. Concurrent with the decline of sea lions and expansion of groundfish fisheries in the Aleutians in the late 1970s, there was a substantial change in ocean climate and declines in the abundance of non-fished species such as capelin, skates and benthic invertebrates [88]. Thus while commercial fisheries might be evoked to partially

explain the interannual fluctuations in the abundance of some species [89], the geographic and temporal coherence of the collapse of large numbers of taxa argues for a large-scale common cause such as changes in ocean climate [45,89–91].

It is important to note that our study only considered the period 2000–2008. Thus, the distribution of fishing effort considered reflects conservation measures implemented since 1990 such as restricting trawl fishing within either 10 or 20 nautical miles (18.5 or 37 km) of rookeries. In addition to fishery restrictions imposed in 1992, 1994 and 2001, there were also major shifts in oceanographic conditions in 1988/89 and 2007/08 that could have altered the relative abundances and availability of prey [92,93]. It is thus difficult, if not impossible, to separate the effects of management actions from the natural effects of ocean climate changes on the prey base of Steller sea lions [7,19,45]. Long-lived species such as sea lions are especially sensitive to anthropogenic effects and environmental changes as they are of large size, high adult survival and low fecundity [94]. The effects of such changes on the demographic parameters of the species can surface years later [95]. Long term monitoring programs to assess the population trends, breeding parameters and survival rates of Steller sea lions should therefore be continued even though sea lion population trends and accessible prey biomass or catch during our study period appeared to be unrelated.

Conclusions

We found little evidence to support the hypothesis that the walleye pollock, Pacific cod and Atka mackerel fisheries in the Aleutian Islands, Bering Sea and Gulf of Alaska modified the abundance and distribution of prey to the detriment of sea lions from 2000–2008. The variable trajectories of sea lion populations appeared to be unrelated to the biomass of groundfish accessible near rookeries, and trends in sea lion numbers were similar with or without fishery removals. These results suggest that sea lions were not limited by groundfish prey and that their populations were largely unaffected by fishery removals of these species during this period. While it is conceivable that these fisheries may have affected sea lions in the past, further constraining groundfish fisheries is unlikely to produce any significant increases in sea lion numbers.

Acknowledgments

We thank Brian Fadely for the sea lion telemetry data, Gina K. Himes Boor for the Platform of Opportunity sightings analysis, Ruth Joy for help with statistical analysis and Edward Richardson, Bill Wilson, Jeannie Heltzel, Phil Dang and Todd Loomis for catch information. Financial support was provided by NOAA to the North Pacific Universities Marine Mammal Research Consortium through the North Pacific Marine Science Foundation.

Author Contributions

Conceived and designed the experiments: TCYH EJG AWT. Performed the experiments: TCYH. Analyzed the data: TCYH. Contributed reagents/materials/analysis tools: TCYH RG. Wrote the paper: TCYH EJG AWT.

References

1. Braham HW, Everitt RD, Rugh DJ (1980) Northern sea lion population decline in the eastern Aleutian Islands. Journal of Wildlife Management 44: 25–33.
2. Hanna S (2000) Setting the fishery management stage: evolution of west coast groundfish management. In: Johnston RS, editor: International Institute of Fisheries Economics and Trade, Corvallis, Oregon, USA.

3. Megrey BA, Westpestad VG (1990) Alaskan groundfish resources: 10 years of management under the Magnuson fishery conservation and management act. North American Journal of Fisheries Management 10: 125–143.
4. Loughlin TR, Merrick RL (1989) Comparison of commercial harvest of walleye pollock and northern sea lion abundance in the Bering Sea and Gulf of Alaska. Proceedings of the International Symposium on the Biology and Management of walleye pollock: University of Alaska Sea Grant Report 89–01.
5. Merrick RL, Loughlin TR, Calkins DG (1987) Decline in abundance in the northern sea lion, *Eumetopias jubatus* in Alaska, 1956–86. Fishery Bulletin 85: 351–365.
6. Springer AM (1992) A review: walleye pollock in the North Pacific—how much difference do they really make? Fisheries oceanography 1: 80–96.
7. Bernard DR, Jeffries SJ, Knapp G, Trites AW (2011) An independent, scientific review of the Biological Opinion (2010) of the National Marine Fisheries Service Fisheries Management Plan for the Bering Sea/Aleutian Islands management areas. Alaska Department of Fish and Game, Special Publication 11–16, Anchorage, AK, 136 pp. pp. 136.
8. Alverson D (1992) A review of commercial fisheries and the Steller sea lion (*Eumetopias jubatus*): the conflict arena. Reviews in Aquatic Sciences 6: 203–256.
9. Alverson DL (1991) Commercial fisheries and the Steller sea lion (*Eumetopias jubatus*): the conflict arena. Fisheries Research Institute, University of Washington, Seattle, Washington, USA.
10. Lowry LF (1982) Documentation and assessment of marine mammal-fishery interactions in the Bering Sea. In: Sobol K, editor. 47th North American Wildlife Conference, Bering Sea interactions. Wildlife Management Institute, Washington, D.C., USA.
11. Lowry LF (1986) Sizes of walleye pollock, *Theragra chalcogramma*, consumed by marine mammals in the Bering Sea. Fishery Bulletin 84: 192–197.
12. Zeppelin TK, Tollit DJ, Call KA, Orchard TJ, Gudmundson CJ (2004) Sizes of walleye pollock (*Theragra chalcogramma*) and Atka mackerel (*Pleurogrammus monopterygius*) consumed by the western stock of Steller sea lions (*Eumetopias jubatus*) in Alaska from 1998 to 2000. Fishery Bulletin 102: 509–521.
13. Trites AW, Donnelly CP (2003) The decline of Steller sea lions in Alaska: a review of the nutritional stress hypothesis. Mammal Review 33: 3–28.
14. Trites AW, Larkin PA (1992) The status of Steller sea lion populations and the development of fisheries in the Gulf of Alaska and Aleutian Islands: Report to the Pacific States Marine Fisheries Commission. University of British Columbia, Fisheries Centre Report. 134 p.
15. Ferrero RC, Fritz LW (1994) Comparisons of walleye pollock, *Theragra chalcogramma*, harvest to Steller sea lion, *Eumetopias jubatus*, abundance in the Bering Sea and Gulf of Alaska. U.S. Department of Commerce, NOAA technical memorandum, NMFS-AFSC-43. 25 p.
16. Sampson DB (1995) An analysis of groundfish fishing activities near Steller sea lion rookeries in Alaska. Oregon State University, Hatfield Marine Science Center, Newport, Oregon. pp. 40.
17. Trites AW, Money J, Larkin PA (1999) The decline of Steller sea lions (*Eumetopias jubatus*) and the development of commercial fisheries in the Gulf of Alaska and Aleutian Islands from 1950 to 1990. Unpublished report, Marine Mammal Research Unit, Fisheries Centre, University of British Columbia.
18. Dillingham PW, Skalski JR, Ryding KE (2006) Fine-scale geographic interations between Steller sea lion (*Eumetopias jubatus*) trends and local fisheries. Canadian Journal of Fisheries and Aquatic Sciences 63: 107–119.
19. Hennen D (2006) Associations between the Alaska Steller sea lion decline and commercial fisheries. Ecological Applications 16: 704–717. PMID: 16711057
20. Calkins DG (2008) Fixed gear-marine mammal interactions study. North Pacific Wildlife Consulting, Texas A&M University Research Foundation.
21. Baraff LS, Loughlin TR (2000) Trends and potential interactions between pinnipeds and fisheries of New England and the U.S. west coast. Marine Fisheries Review 62: 1–39.
22. Sease JL, Taylor W, Loughlin TR, Pitcher KW (2001) Aerial and land-based surveys of Steller sea lions (*Eumetopias jubatus*) in Alaska, June and July 1999 and 2000.: U.S. Department of Commerce, NOAA Technical Memo NMFS-AFSC-122.
23. Shima M, Hollowed AB, VanBlaricom GR (2002) Changes over time in the spatial distribution of walleye pollock (*Theragra chalcogramma*) in the Gulf of Alaska, 1984–1996. Fishery Bulletin 100: 307–323.
24. Conners ME, Munro P (2008) Effects of commercial fishing on local abundance of Pacific cod (*Gadus macrocephalus*) in the Bering Sea. Fishery Bulletin 106: 281–292.
25. Conn PB, Johnson DS, Fritz LW, Fadely BS (2013) Use and misuse of fishery and survey data to detect prey removal effects on Steller sea lions (*Eumetopias jubatus*). Draft manuscript submitted to the Alaska Regional office of NOAA Fisheries. 49 pp.

26. Guenette S, Heymans SJJ, Christensen V, Trites AW (2006) Ecosystem models show combined effects of fishing, predation, competition and ocean productivity on Steller sea lions (*Eumetopias jubatus*) in Alaska. Canadian Journal of Fisheries and Aquatic Sciences 63: 2495–2517.
27. Cornick LA, Neill W, Grant WE (2006) Assessing competition between Steller sea lions and the commercial groundfishery in Alaska: A bioenergetics modelling approach. Ecological Modelling 199: 107–114.
28. Merrick RL, Loughlin TR (1997) Foraging behavior of adult female and young-of-year Steller sea lions in Alaskan waters. Canadian Journal of Zoology 75: 776–786.
29. Merrick RL, Loughlin TR, Antonelis GA, Hill R (1994) Use of satellite linked telemetry to study Steller sea lion and northern fur seal foraging. Polar research 13: 105–114.
30. Raum-Suryan KL, Rehberg MJ, Pendleton GW, Pitcher KW, Gelatt TS (2004) Development of dispersal, movement patterns, and haul-out use by pup and juvenile Steller sea lions (*Eumetopias jubatus*) in Alaska. Marine Mammal Science 20: 823–850.
31. Fadely BS, Robson BW, Sterling JT, Greig A, Call KA (2005) Immature Steller sea lion (*Eumetopias jubatus*) dive activity in relation to habitat features of the eastern Aleutian Islands. Fisheries Oceanography 14: 243–258.
32. Rehberg MJ, Andrews RD, Swain UG, Calkins DG (2009) Foraging behavior of adult female Steller sea lions during the breeding season in Southeast Alaska. Marine Mammal Science 25: 588–604.
33. Rehberg MJ, Burns JM (2008) Differences in diving and swimming behavior of pup and juvenile Steller sea lions (*Eumetopias jubatus*) in Alaska. Canadian Journal of Zoology 86: 539–553.
34. Winter A, Foy RJ, Wynne KM (2009) Seasonal difference in prey availability around a Steller sea lion haulout and rookery in the Gulf of Alaska. Aquatic Mammals 35: 145–162.
35. Sinclair EH, Johnson DS, Zeppelin TK, Gelatt TS (2013) Decadal variation in the diet of western stock Steller sea lions (*Eumetopias jubatus*). U.S. Department of Commerce, NOAA Technical Memorandum NMFS-AFSC-248, 67 pp.
36. Winship AJ, Trites AW (2006) Risk of extirpation of Steller sea lions in the Gulf of Alaska and Aleutian Islands: A population viability analysis based on alternative hypotheses for why sea lions declined in western Alaska. Marine Mammal Science 22: 124–155.
37. Gryba R, Gregr EJ, Joy R, Trites AW (2014) Predicted distributions and biomass of walleye pollock, Pacific cod and Atka mackerel within Steller sea lion critical habitat in Alaska. Fisheries Centre Working Paper 2014-29, The University of British Columbia, Vancouver, B.C., Canada.
38. R Development Core Team (2009) R: A Language and Environment for Statistical Computing. R Foundation for Statistical Computing, Vienna.
39. Pinheiro JC, Bates DM (2000) Mixed-Effects Models in S and S-PLUS. New York: Springer.
40. Ladd C, Hunt GL Jr, Mordy CW, Salo SA, Stabeno PJ (2005) Marine environments of the eastern and central Aleutian Islands. Fisheries oceanography 14: 22–38.
41. Call KA, Loughlin TR (2005) An ecological classification of Alaskan Steller sea lion (*Eumetopias jubatus*) rookeries: a tool for conservation/management. Fisheries Oceanography 14: 212–222.
42. Hunt GL Jr, Stabeno PJ (2005) Oceanography and ecology of the Aleutian Archipelago: spatial and temporal variation. Fisheries oceanography 14: 292–306.
43. Logerwell EA, Aydin K, Barbeaux S (2005) Geographic patterns in the demersal ichthyofauna of the Aleutian Islands shelf. Fisheries Oceanography 14: 93–112.
44. Sinclair EH, Moore SE, Friday NA, Zeppelin TK, Waite JM (2005) Do patterns of Steller sea lion (*Eumetopias jubatus*) diet, population trend and cetacean occurrence reflect oceanographic domains from the Alaska Peninsula to the central Aleutian Islands? Fisheries oceanography 14: 223–242.
45. Trites AW, Miller AJ, Maschner HDG, Alexander MA, Bograd SJ, Calder JA, et al. (2007) Bottom-up forcing and the decline of Steller sea lions (Eumetopias jubatus) in Alaska: assessing the ocean climate hypothesis. Fisheries Oceanography 16: 46–67.
46. NMFS (2008) Percent observer coverage in the Bering Sea, Aleutian Islands, and Gulf of Alaska, 2004–2007. NMFS Alaska Region, Juneau, Alaska.
47. Trites AW, Porter BT (2002) Attendance patterns of Steller sea lions (*Eumetopias jubatus*) and their young during winter. Journal of Zoology 256: 547–556.
48. Marcotte ML (2006) Steller watch: Timing of weaning and seasonal patterns in numbers and activities of Steller sea lions (*Eumetopias jubatus*) at a year-round haulout site in southeast Alaska: MSc thesis, University of British Columbia, Department of Zoology. 82 p.
49. Trites AW, Porter BP, Deecke VB, Coombs AP, Marcotte ML, Rosen DAS (2006) Insights into the timing of weaning and the attendance patterns of lactating Steller sea lions (*Eumetopias jubatus*) in Alaska during winter, spring and summer. Aquatic Mammals 32: 85–97.

50. Eastman JR (2001) IDRISI Release 2-Guide to GIS and image processing, 32.20 edition.: Clark University, Worcester, MA. doi: 10.1056/NEJMoa1300955 PMID: 25014686
51. Rea LD, Castellini MA, Fadely BS, Loughlin TR (1998) Health status of young Alaska Steller sea lion pups (*Eumetopias jubatus*) as indicated by blood chemistry and hematology. Comparative Biochemistry and Physiology Part A 120: 617–623.
52. Loughlin TR, Sterling JT, Merrick RL, Sease JL, York AE (2003) Immature Steller sea lion diving behavior. Fishery Bulletin 101: 566–582.
53. Sinclair E, Zeppelin T (2002) Seasonal and spatial differences in diet in the western stock of steller sea lions (*Eumetopias jubatus*). Journal of Mammalogy 83: 973–990.
54. Begon M, Townsend CR, Harper JL (2006) Ecology: From individuals to Ecosystems: Blackwell, Oxford, UK.
55. Kane A, Jackson AL, Monadjem A, Colomer MA, Margalida A (2015) Carrion ecology modelling for vulture conservation: are vulture restaurants needed to sustain the densest breeding population of the African White-backed Vulture? Animal Conservation.
56. Margalida A, Colomer MA, Sanuy D (2011) Can wild ungulate carcasses provide enough biomass to maintain avian scavenger populations? An empirical assessment using a bio-inspired computational model. PLoS ONE 6: e20248. doi: 10.1371/journal.pone.0020248 PMID: 21629647
57. Margalida A, Colomer MÀ (2012) Modelling the effects of sanitary policies on European vulture conservation. Sci Rep 2: 753. doi: 10.1038/srep00753 PMID: 23082243
58. Gregr EJ, Trites AW (2008) A novel presence-only validation technique for improved Steller sea lion *Eumetopias jubatus* critical habitat descriptions. Marine Ecology Progress Series 365: 247–261.
59. Matthiopoulos J (2003) The use of space by animals as a function of accessibility and preference. Ecological Modelling 159: 239–268.
60. Sigler MF, Tollit DJ, Vollenweider JJ, Thedinga JF, Csepp DJ, Womble JN, et al. (2009) Steller sea lion foraging response to seasonal changes in prey availability. Marine Ecology Progress Series 388: 243–261.
61. Sigler MF, Csepp DJ (2007) Seasonal abundance of two important forage species in the North Pacific Ocean, Pacific herring and walleye pollock. Fisheries Research 83: 319–331.
62. Kitts DD, Huynh MD, Hu C, Trites AW (2004) Season variation in nutrient composition of Alaskan walleye pollock. Canadian Journal of Zoology 82: 1408–1415.
63. Sinclair EH, Johnson DS, Zeppelin TK, Gelatt TS (2013) Decadal variation in the diet of Western Stock Steller sea lions (*Eumetopias jubatus*). U.S. Dep. Commer., NOAA Tech. Memo. NMFSAFSC-248, 67 pp.
64. Pitcher KW (1981) Prey of the Steller sea lion, *Eumetopias jubatus*, in the Gulf of Alaska. Fishery Bulletin 79: 467–472.
65. Calkins DG (1998) Prey of Steller sea lions in the Bering Sea. Biosphere Conservation 1: 33–44.
66. Smith R, Paul A, Paul J (1990) Seasonal changes in energy and the energy cost of spawning of Gulf of Alaska Pacific cod. Journal of Fish Biology 36: 307–316.
67. Shimada AM, Kimura DK (1994) Seasonal movements of Pacific cod, *Gadus macrocephalus*, in the eastern Bering Sea and adjacent waters based on tag-recapture data. Fishery Bulletin 92: 800–816.
68. Livingston PA (1993) Importance of predation by groundfish, marine mammals and birds on walleye pollock *Theragra chalcogramma* and Pacific herring *Clupea harengus* in the eastern Bering Sea. Marine Ecology Progress Series 102: 205–215.
69. NMFS (2009) Stock assessment and fishery evaluation report for the groundfish fisheries of the Gulf of Alaska and Bering Sea/Aleutian Islands area: Economic status of the groundfish fisheries off Alaska. AFSC, Seattle, Washington.
70. Lander ME, Fritz LW, Johnson DS, Logsdon MG (2013) Population trends of Steller sea lions (*Eumetopias jubatus*) with respect to remote sensing measures of chlorophyll-*a* in critical habitat. Marine Biology 160: 195–209.
71. Lander ME, Loughlin TR, Logsdon MG, VanBlaricom GR, Fadely BS, Fritz LW (2009) Regional differences in the spatial and temporal heterogeneity of oceanographic habitat used by Steller sea lions. Ecological Applications 19: 1645–1659. PMID: 19769109
72. Orians GH, Pearson NE (1979) On the theory of central place foraging. In: Horn DJ, Mitchell RD, Stairs GR, editors. Analysis of ecological systems: Ohio University Press, pp. 154–177.
73. Brandon EAA (2000) Maternal investment in Steller sea lions in Alaska. [PhD thesis]. Galveston, TX: Texas A&M University.
74. Milette LL, Trites AW (2003) Maternal attendance patterns of lactating Steller sea lions (*Eumetopias jubatus*) from a stable and a declining population in Alaska. Canadian Journal of Zoology 81: 340–348.

75. Ban S, Trites AW (2007) Quantification of terrestrial haul-out and rookery characteristics of Steller sea lions. Marine Mammal Science 23: 496–507.
76. Karpouzi VS, Watson R, Pauly P (2007) Modelling and mapping resource overlap between seabirds and fisheries on a global scale: a preliminary assessment. Marine Ecology Progress Series 343: 87–99.
77. Austin D, Bowen WD, McMillan JI (2004) Intraspecific variation in movement patterns: modeling individual behaviour in a large marine predator. Oikos 105: 15–30.
78. Edwards AM, Phillips RA, Watkins NW, Freeman MP, Murphy EJ, Afanasyev V, et al. (2007) Revisiting Levy flight search patterns of wandering albatrosses, bumblebees and deer. Nature 449: 1044–1049. PMID: 17960243
79. Viswanathan GM, Afanasyev V, Buldyrev SV (1996) Levy flight search patterns of wandering albatrosses. Nature 381: 413–415.
80. Viswanathan GM, Sergey V, Buldyrev SV, Havlin S, da Luz MGE, Raposo EP, et al. (1999) Optimizing the success of random searches. Nature 401: 911–914. PMID: 10553906
81. McDermott SF, Fritz LW, Heist V (2005) Estimating movement and abundance of Atka mackerel (*Pleurogrammus monopterygius*) with tag-release-recapture data. Fisheries Oceanography 14(supp.1): 113–130.
82. Winship AJ, Trites AW (2003) Prey consumption of Steller sea lions (*Eumetopias jubatus*) off Alaska: How much prey do they require? Fishery Bulletin 101: 147–167.
83. Womble JN, Sigler MF (2006) Seasonal availability of abundant, energy-rich prey influences the abundance and diet of a marine predator, the Steller sea lion *Eumetopias jubatus* Marine Ecology Progress Series 325: 281–293.
84. Winship AJ, Trites AW, Rosen DAS (2002) A bioenergetic model for estimating the food requirements of Steller sea lions (*Eumetopias jubatus*) in Alaska. Marine Ecology Progress Series 229: 291–312.
85. Bowen WD, Harwood J, Goodman D, Swartzman GL (2001) Review of the November 2000 Biological Opinion and Incidental Take Statement with respect to the western stock of the Steller sea lion. Final Report to the North Pacific Fisheries Management Council, May 2001. 19 p.
86. NRC (2003) Decline of the Steller sea lion in Alaskan waters; untangling food webs and fishing nets. National Academy press, Washington, D.C. 184 p.
87. Loughlin TR, York AE (2000) An accounting of the sources of Steller sea lion mortality. Marine Fisheries Review 62: 40–45.
88. Mueter FJ, Norcross BL (2002) Spatial and temporal patterns in the demersal fish community on the shelf and upper slope regions of the Gulf of Alaska. Fishery Bulletin 100: 559–581.
89. Orensanz JM, Armstrong J, Armstrong D, Hilborn R (1998) Crustacean resources are vulnerable to serial depletion—the multifaceted decline of crab and shrimp fisheries in the greater Gulf of Alaska. Reviews in Fish Biology and Fisheries 8: 117–176.
90. Anderson PJ, Piatt JF (1999) Community reorganization in the Gulf of Alaska following ocean climate regime shift. Marine Ecology Progress Series 189: 117–123.
91. Maschner HDG, Trites AW, Reedy-Maschner KL, Betts M (2013) The decline of Steller sea lions (Eumetopias jubatus) in the North Pacific: insights from indigenous people, ethnohistoric records and archaeological data. Fish and Fisheries: doi: 10.1111/faf.12038
92. NMFS (2014) Endangered Species Act. Section 7 Consultation Biological Opinion on authorization of Alaska groundfish fisheries under the proposed revised Steller sea lion protection measures. Silver Spring, Maryland. 453 (+Appendices) pp.
93. Litzow MA, Mueter FJ (2014) Assessing the ecological importance of climate regime shifts: An approach from the North Pacific Ocean. Progress in Oceanography 120: 110–119.
94. Bennett PM, Owens IPF (1997) Variation in extinction risk among birds: chance or evolutionary predisposition? Proceedings of the Royal Society B 264: 401–408.
95. Margalida A, Colomer MA, Oro D (2014) Man-induced activities modify demographic parameters in a long-lived species: effects of poisoning and health policies. Ecological Applications 24: 436–444. PMID: 24834731
